# Efficacy and Safety of a Fixed-Dose Combination of Vildagliptin and Pioglitazone in Indian Patients With Type 2 Diabetes Mellitus: A Randomized, Open-Label, Comparative, Phase III Study

**DOI:** 10.7759/cureus.44548

**Published:** 2023-09-01

**Authors:** Suhas Erande, Jotideb Mukhopadhyay, Amol Dange, Anushka Deogaonkar, Ashish Birla, Chetan Doshi, Santosh Revankar, Sridhar S B, Neeraj Kumar, Pramod V Kadam

**Affiliations:** 1 Diabetes and Endocrinology, Akshay Hospital & Diabetic Speciality Centre, Pune, IND; 2 General Medicine, Institute of Post Graduate Medical Education & Research, Kolkata, IND; 3 Medicine, LifeTree Hospital, Pune, IND; 4 Medicine, Akshay Hospital, Pune, IND; 5 Scientific Services, USV Private Limited, Mumbai, IND; 6 Research and Development (R&D), USV Private Limited, Mumbai, IND; 7 Clinical Research, USV Private Limited, Mumbai, IND

**Keywords:** pioglitazone, vildagliptin, t2dm, ppg, hba1c, fpg, fixed-dose combination

## Abstract

Background

Type 2 diabetes mellitus (T2DM) arises due to a range of pathological abnormalities, necessitating a combination therapy to achieve optimal glycemic control. Vildagliptin, an effective and selective DPP-4 inhibitor, and pioglitazone, an insulin sensitizer, offer distinct mechanisms of action. Hence, the integration of these medications represents a logical and justified therapeutic strategy

Objective

To compare the efficacy, safety, and tolerability of vildagliptin and pioglitazone 50 mg/15 mg fixed-dose combination (FDC) tablets with individual monotherapy vildagliptin 50 mg and pioglitazone 15 mg tablets in Indian T2DM patients who were inadequately controlled on metformin monotherapy.

Methods

This was a randomized, open-label, comparative, multicenter, phase III study involving 195 T2DM patients with inadequate glycemic control on metformin ≥ 1000 mg/day. Patients were randomly assigned in a 1:1:1 ratio to the test product group (n=65) (vildagliptin 50 mg + pioglitazone 15 mg FDC tablets), reference product group 1 (n=65) (vildagliptin 50 mg tablet), or reference product group 2 (n=65) (pioglitazone 15 mg tablet reference product). The primary endpoint was the mean change in HbA1c levels from baseline to end of the study visit (12 weeks (84 days ±2)). The secondary endpoints were the mean change in fasting plasma glucose (FPG) and 2-hr postprandial plasma glucose (2-hr PPG) levels. Safety parameters were assessed till the end of the study.

Results

A total of 178 patients completed the study. At 12 weeks, the mean HbA1c level in the test group reduced to 6.85 ± 1.27%, in the reference product 1 group to 7.56 ± 1.72%, and in the reference product 2 groups to 7.37 ± 1.59%. The mean change in Hb1Ac from baseline in the test group was statistically significant compared to the reference groups (p=0.037). Similarly, the mean changes in the FPG and 2hr-PPG with the test product were statistically significant compared to reference products (p=0.041). The adverse events were comparable across all the treatment groups.

Conclusion

In Indian T2DM patients inadequately controlled on a daily maximum dose of metformin, treatment with vildagliptin and pioglitazone FDC showed better glycemic control than either vildagliptin or pioglitazone along with a good tolerability profile.

## Introduction

In India, 77 million people were estimated to have diabetes in 2019; by 2045, that number is projected to reach over 134 million. Type 2 diabetes mellitus (T2DM), constituting 90% of all cases of diabetes, has become a significant cause of disability and death, affecting even the younger age groups. Individuals with T2DM can suffer multiorgan complications, leading to an increased rate of premature morbidity and mortality, reducing their life expectancy and imposing a profound economic burden on the Indian healthcare system [1].

Several underlying defects, viz., insulin resistance, impairment in pancreatic α-cells and β-cells functions, and renal glucose reabsorption, contribute to the development of T2DM [2]. Thus, individuals with T2DM often require multiple pharmaceutical combinations to achieve their treatment objectives. Initial combination therapy might offer better and longer-lasting glycemic control and allow lower component doses, thus minimizing dose-related adverse events [3]. The landmark UK Prospective Diabetes Study reported that monotherapy did not provide long-term stable glycemic control, requiring the addition and a combination of glucose-lowering agents. Early combination therapy using agents with complementary modes of action holds the promise of altering the course of the disease, thereby providing extended periods of stable HbA1c levels, delaying the need for therapy intensification, and reducing the risk of chronic complications [4]. Studies have also indicated that tight glycemic control may prevent histological progression in nonalcoholic fatty liver disease in T2DM patients [5].

In this regard, a combination therapy involving a thiazolidinedione (TZD) and DPP-4 inhibitor appears attractive. TZDs are the only antidiabetic medications that primarily work as an insulin sensitizer in peripheral and hepatic tissues by interacting with and activating the nuclear peroxisome proliferator-activated receptor (PPAR) in those tissues [6]. Pioglitazone also has beneficial effects on lipid metabolism and cardiovascular (CV) risk [7].

Pioglitazone significantly improves liver histology in patients with nonalcoholic fatty liver disease, such as steatosis, inflammation, and ballooning. Furthermore, pioglitazone can improve plasma aspartate aminotransferase (AST), alanine transaminase (ALT), and other liver biological indicators [8].

Vildagliptin is a potent and selective DPP-4 inhibitor. It enhances pancreatic islet function, as shown by an improvement in α- and β-cells sensitivity to glucose after treatment. Vildagliptin also reduces liver glucose production before and after meals and throughout the nocturnal postabsorptive period. Vildagliptin's clinical utility as an antidiabetic agent for treating T2DM has been established by its efficacy profile, combined with a low risk of hypoglycemia, no weight gain, and no increased risk of CV events [9].

Fixed-dose combinations (FDCs) are essential in achieving glycemic targets in T2DM. When used rationally, FDCs offer numerous benefits, such as low pill burden, low risk of side effects, and good patient compliance, all of which contribute to improved efficacy [10]. According to available clinical data, combining vildagliptin with pioglitazone is a practical therapeutic approach in patients with T2DM who are uncontrolled on monotherapy or cannot tolerate metformin or sulfonylurea [11]. Therefore, an FDC of vildagliptin and pioglitazone appears to be a promising and clinically beneficial therapeutic option for managing T2DM.

The present clinical trial was conducted to ascertain the efficacy, safety, and tolerability of vildagliptin and pioglitazone FDC in Indian patients with T2DM inadequately controlled on metformin monotherapy.

## Materials and methods

Study design

**Figure 1:**
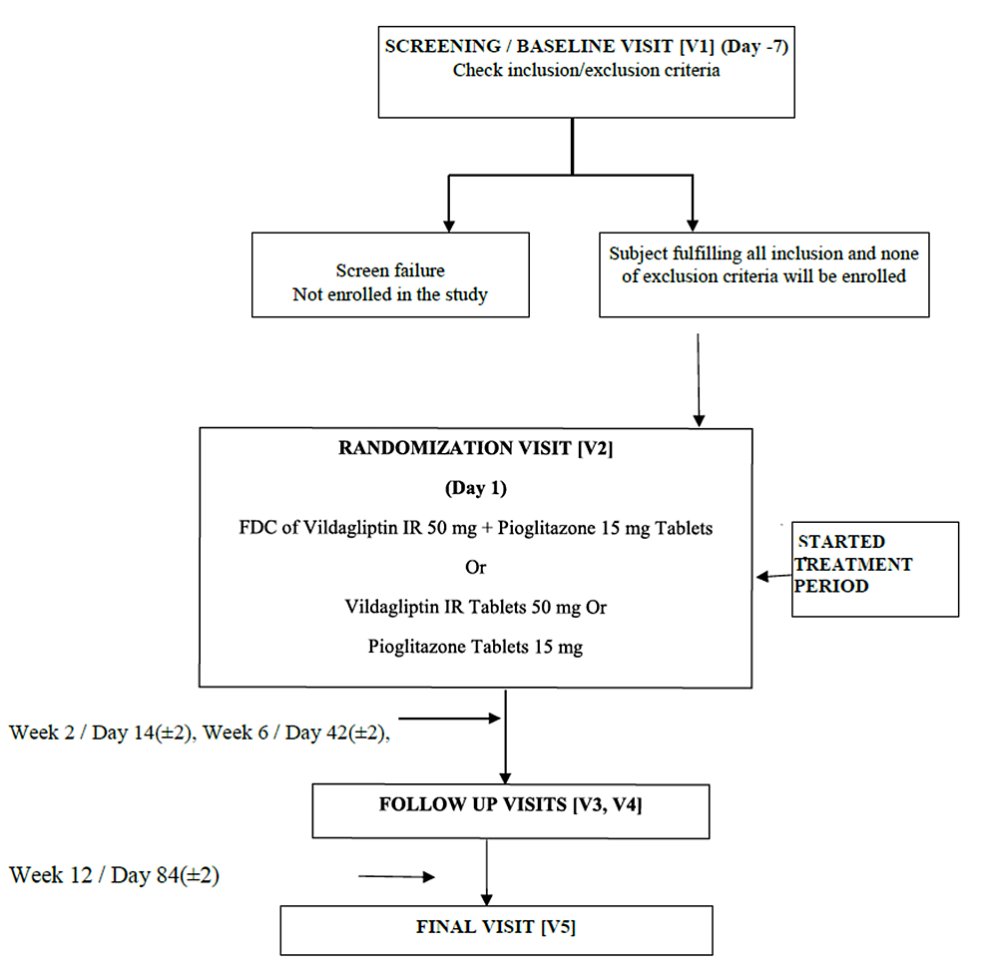
Study flowchart

This was a phase III, prospective, randomized, open-label, comparative, parallel-group, multicenter clinical study. All potential study participants underwent a single screening visit during which the inclusion/exclusion criteria were evaluated. At the randomization visit (day one), the subjects were randomized to receive either the test product, i.e., vildagliptin and pioglitazone hydrochloride 50 mg/15 mg FDC tablets, or reference products, vildagliptin 50 mg tablets, and pioglitazone hydrochloride 15 mg tablets. Follow-up visits were conducted on week one/day 14(±2), week six/day 42 (±2), and week 12/day 84 (±2) (final visit) of treatment to assess efficacy, safety, and tolerability. Subjects were provided with a diary at the randomization visit along with a glucometer to record details about study drug administration, adverse events, and self-monitored blood glucose levels. Subjects are required to bring a completed diary at each visit (Figure 1).

Study population

The study enrolled patients who had been on the treatment with metformin ≥1000 mg/day for at least three months before screening and had inadequate glycemic control (glycosylated hemoglobin (HbA1c) levels of ≥ 7% to ≤ 10%). The study medication was administered twice daily for 12 weeks. Additionally, patients continued to receive metformin at stable doses of ≥ 1000 mg/day throughout the study period in an open-label manner (12 weeks). The mean age of patients in the test product group was 51.31 (SD: 9.78) years; in reference, product 1 group was 49.82 (9.45) years, and, in reference, product 2 group was 49.20 (9.59) years.

Patients were excluded if they had a history of type 1 diabetes or secondary forms of diabetes or diabetes insipidus, metabolic acidosis or diabetic ketoacidosis, body mass index (BMI) ≥ 45.0 kg/m2, congestive heart failure, transient ischemic attack, unstable or previously undiagnosed arrhythmia, cardiac surgery or revascularization (coronary angioplasty or bypass grafts), or cerebrovascular accident, uncontrolled hypertension (sitting systolic BP ≥ 160 mmHg and diastolic BP ≥ 100 mmHg), clinically significant 12-lead ECG abnormality, liver diseases (hepatitis B, hepatitis C), or HIV infection. Patients with an estimated glomerular filtration rate (eGFR) <60 mL/min/1.73 m2 (using the modification of diet in renal disease (MDRD) equation) were excluded. Patients with any of the following laboratory abnormalities were also excluded: fasting plasma glucose (FPG) > 220 mg/dL at screening and even after one week of repeat tests, ALT or AST greater than three times the upper limit of normal (ULN) and total bilirubin > 1.5 times the ULN.

Study objective

The primary objective of the study was to evaluate the efficacy of the test product, i.e., vildagliptin, and pioglitazone hydrochloride 50 mg/15 mg FDC tablets, with the reference products, i.e., vildagliptin 50 mg tablets, and pioglitazone hydrochloride 15 mg tablets administered as monotherapy. The secondary objective was to evaluate the safety and tolerability of the test product compared with the reference products.

Efficacy endpoints

The primary efficacy endpoint was the mean change in HbA1c levels from baseline to the end of the study visit (12 weeks (84 days ±2)). Secondary endpoints were the mean change in FPG and 2-hr postprandial plasma glucose (2-hr PPG) levels from baseline to the end of the study visit (12 weeks (84 days ±2)). The PROC MIXED model evaluated the mean change in HbA1c, FPG, and 2-hr PPG.

Safety endpoints

Safety monitoring included the frequency of hypoglycemia, adverse events, laboratory values including hematology, biochemistry, and urinalysis, vital signs, 12-lead ECG, and physical examination.

Adverse events were classified as mild (transient discomfort and does not interfere in a significant manner with the subject and resolves spontaneously or may require minimal therapeutic intervention), moderate (produces limited impairment of function and may require therapeutic intervention but produces no sequelae), and severe (results in a marked impairment of function, may lead to a temporary inability to resume the usual life pattern, and produces sequelae, which require (prolonged) therapeutic intervention).

Data analysis

All analyses were performed using Statistical Analysis Software (SAS®) Version 9.4 or above. The primary and secondary efficacy variables were analyzed using the modified intention-to-treat (mITT) analysis set. The mITT analysis set included all randomized subjects who received at least one dose of study medication and had a non-missing baseline measurement for the efficacy variable. In addition, a supportive analysis was performed for the primary efficacy endpoint using the per-protocol set (PPS). The PPS included all randomized subjects who received at least one dose of study medication, completed the study, and had no major protocol deviations. Changes from baseline in primary and secondary endpoints were analyzed using the analysis of covariance model (ANCOVA), which included the treatment group, sex, age, and baseline variables as covariates. The ANCOVA model estimated the least squares mean (LSM) of products and their 95% confidence interval (CI). The safety analysis set included all randomized subjects who received at least one dose of study medication.

Ethics and good clinical practice

All participants provided written informed consent. Institutional ethics committees at each study site approved the protocol and documents for informed consent before the study initiation. The study was conducted in accordance with the principles of good clinical practice (GCP), the current version of the Declaration of Helsinki, the Indian Council of Medical Research (ICMR) Ethical Guidelines for Biomedical Research on Human Patients (2017), and applicable regulatory guidelines.

## Results

**Figure 2:**
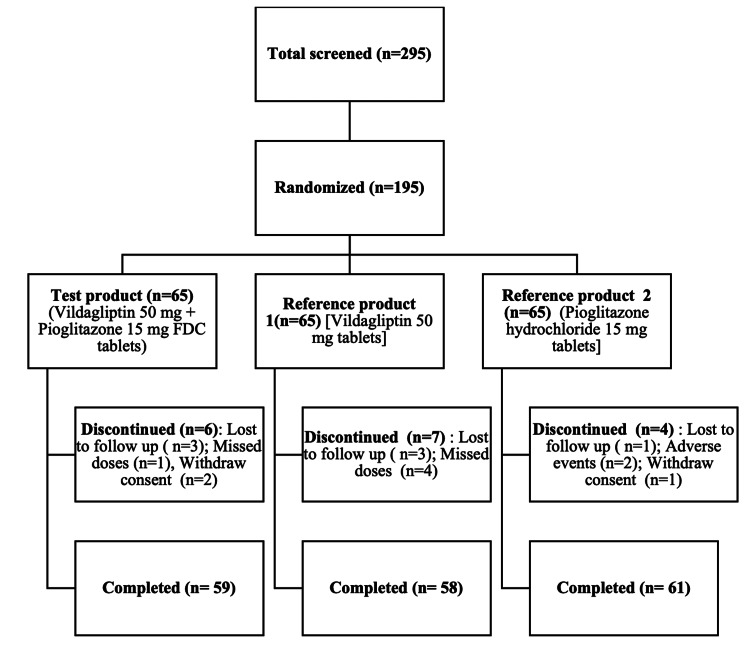
Disposition of patients from screening through study endpoint

In total, 195 subjects participated in the study. In a 1:1:1 ratio, subjects were randomly assigned to the test product group (n = 65) (vildagliptin 50 mg + pioglitazone 15 mg FDC tablets), reference product group 1 (n = 65) (vildagliptin 50 mg tablet), or reference product group 2 (n = 65) (pioglitazone 15 mg tablet reference product). Overall, 178 subjects completed the study, 59 in the test product group, 58 in the reference product group 1, and 61 in the reference product group 2 (Figure 2). 

The mean age of patients in the test product group was 51.31 ± 9.78 years; in the reference product 1 group was 49.82 ± 9.45 years, and in the reference product 2 group was 49.20 ± 9.59 years. All three groups were comparable in demographic and baseline characteristics (Table 1).

**Table 1:** Patient demographics and baseline characteristics BMI: Body mass index; HbA1c: Glycosylated hemoglobin; FPG: Fasting plasma glucose; PPG: Postprandial glucose; SD: Standard deviation; a vildagliptin 50 mg + pioglitazone 15 mg FDC tablets; b vildagliptin 50mg tablets; c pioglitazone 15mg tablets

	Test product^a^ (n=65)	Reference product^b^ (n=65)	Reference product^c^ (n=65)	p-value
Parameter	Mean ± SD	Mean ± SD	Mean ± SD	
Age (years)	51.31 ± 9.78	49.82 ± 9.45	49.20 ± 9.59	0.439
Weight (kg)	66.9± 10.46	68.08 ± 9.72	66.15 ± 11.06	0.572
Height (cm))	160 ± 8.3	159.36± 9.64	161 ± 8.64	0.569
BMI (kg/m^2^)	26.11± 3.37	26.97± 4.46	25.53 ± 3.85	0.111
HbA1c (%)	8.16± 0.86	8.37± 0.90	8.44 ± 0.91	0.167
FPG (mg/dl)	141.34 ± 37.66	136.67± 37.71	140.07± 37.59	0.766
2hr-PPG (mg/dl)	222.22 ± 77.08	228.53 ± 80.20	219.34 ± 82.31	0.802

Efficacy

The primary endpoint was analyzed using the mITT population set. Figure ​3 represents the mean ± standard deviation (SD) changes in HbA1c during a 12-week treatment period in the three treatment arms. At baseline, the mean HbA1c level in the test group was 8.16 ± 0.86%, while in the reference product, product 1 group was 8.37 ± 0.90%, and in the reference product, product 2 group was 8.44 ± 0.91%. At week 12/day 84 (± 2), the mean HbA1c levels in the test group (n = 58), reference product 1 (n = 58), and reference product 2 group (n = 61) were reduced to 6.85 ± 1.27%, 7.56 ± 1.72%, and 7.37 ± 1.59%, respectively. The mean reduction in HbA1c from baseline to endpoint was significantly greater (p = 0.037) in patients receiving test products than reference products. The proportion of patients who achieved HbA1c < 7% was 51.7% in the test group, 41.4% in the reference product 1 group, and 41% in the reference product 2 group. Although the proportion was higher in the test group, the difference across groups was not statistically significant (p = 0.415).

**Figure 3:**
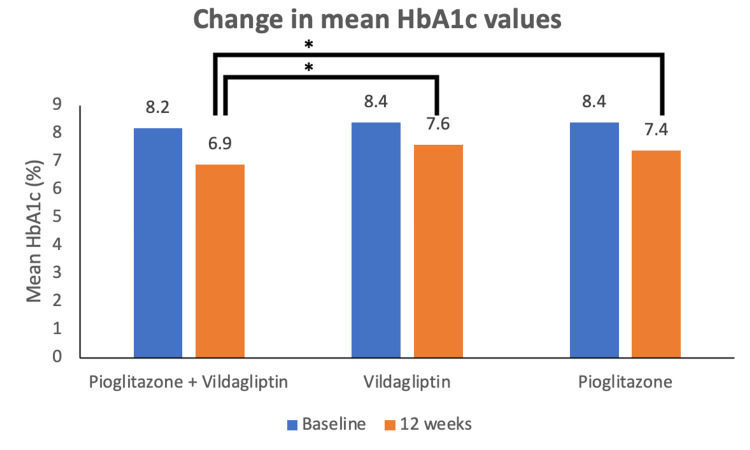
Mean (±SD) values of HbA1c at baseline and after 12 weeks of treatment with test product (vildagliptin 50 mg + pioglitazone 15 mg FDC tablets), reference product 1 (vildagliptin 50 mg tablets) and reference product 2 (pioglitazone hydrochloride 15 mg tablets) *p=0.037 vs. reference products 1 and 2. SD: Standard deviation

The estimate of the difference in the LSM of the test to reference product 1 was -0.6312 (95% CI = -1.2985 to 0.03603) and to reference product 2 was -0.6426 (95% CI = -1.2797 to -0.00546). The upper bound of 95% CI was less than the predefined non-inferiority margin of 0.3, indicating the non-inferiority of the test product over the reference products 1 and 2.

**Figure 4:**
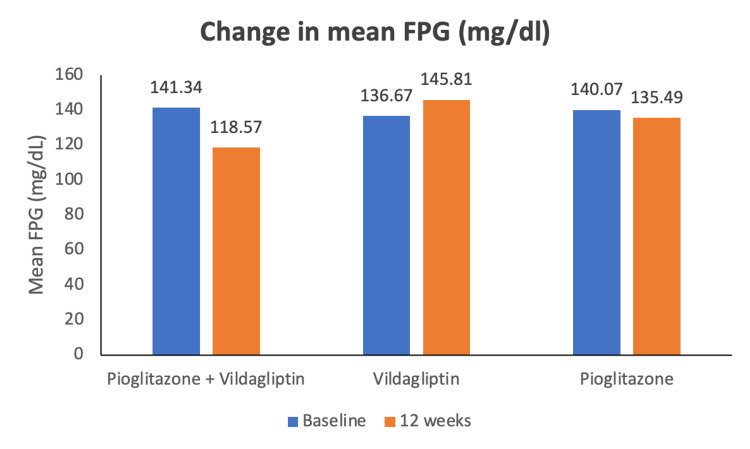
Mean (±SD) values of FPG at baseline and after 12 weeks of treatment with test product (vildagliptin 50 mg + pioglitazone 15 mg FDC tablets), reference product 1 (vildagliptin 50 mg tablets) and reference product 2 (pioglitazone hydrochloride 15 mg tablets) *p=0.041 vs. reference products 1 and 2. FPG: Fasting plasma glucose. SD: Standard deviation.

The secondary efficacy was also analyzed using the mITT population set. Along with the reduction in HbA1c, FPG also decreased to statistically significant levels during the 12-week treatment period (Figure 4). At screening, the mean FPG level in the test group was 141.34 ± 37.66 mg/dl, while in the reference product, product 1 group was 136.67 ± 37.71 mg/dl, and reference product 2 group was 140.07± 37.59 mg/dl. At 12 weeks/day, 84 (± 2), statistically significant changes in mean values were observed in the test product group compared to reference product groups (p =.041). In the test group (n = 59), mean FPG reduced to 118.57 ± 52.66 mg/dl, while in the reference product 1 group (n = 58) to 145.81 ± 71.54 mg/dl, and in the reference product 2 group (n = 60) to 135.49 ± 48.91 mg/dl.

The estimate of the difference in LSM of the test to reference product 1 and reference product 2 was -32.45 (95% CI = -55.61 to -9.29) and -30.30 (95% CI = -52.56 to -8.02), respectively. The difference in mean change in FPG from baseline to end of study between the test product and reference product groups was statistically significant.

**Figure 5:**
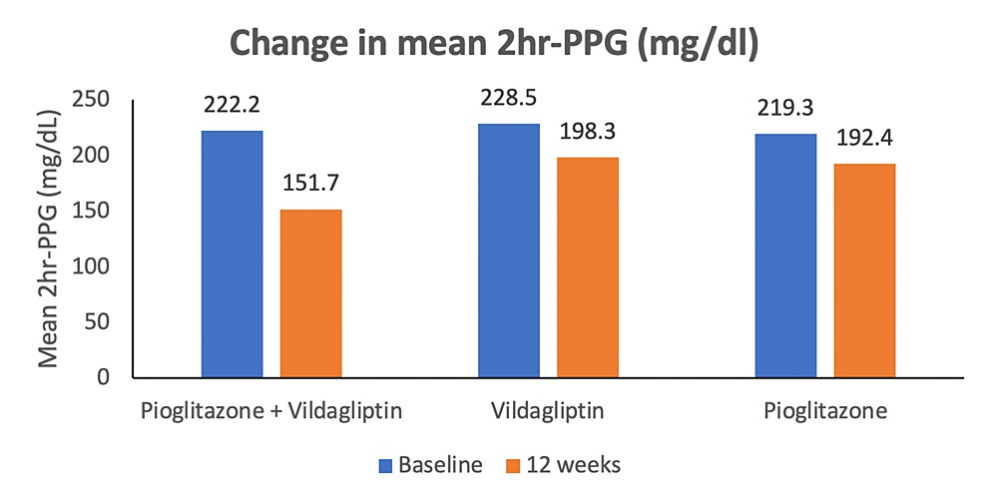
Mean (±SD) values of 2hr-PPG after treatment with test product (vildagliptin 50 mg + pioglitazone 15 mg FDC tablets), reference product 1 (vildagliptin 50 mg tablets) and reference product 2 (pioglitazone hydrochloride 15 mg tablets) *p=0.003 vs. reference products 1 and 2. PPG: Postprandial plasma glucose. SD: Standard deviation.

Mean 2hr-PPG decreased in each treatment group. At baseline, mean 2hr-PPG was 222.22 ± 77.08 mg/dl for the test product group, 228.53 ± 80.20 mg/dl for the reference product 1 group, and 219.34 ± 82.31 mg/dl for reference product 2 groups. At 12 weeks/day, 84 (± 2), it reduced to 151.72± 52.06 mg/dl in the test product group (n = 59) and to 198.27± 91.81 mg/dl, 192.44± 90.59 mg/dl in the reference product 1 (n = 58) and 2 (n = 60) groups, respectively. The mean difference between the test and reference product groups was statistically significant (p = 0.003). Figure 5 depicts the mean (±SD) change in 2hr-PPG across treatment arms during the study period.

The estimate of the difference in LSM of the test to reference product 1 and reference product 2 was - 45.05 (95% CI = -79.58 to -10.50) and -30.30 (95% CI = -57.37 to - 24.37), respectively. The difference in mean change in 2hr-PPG from baseline to end of study between the test product and reference product 1 was statistically significant, while statistically insignificant versus the reference product 2.

Safety and tolerability

Table 2 summarizes the overall adverse events (AEs) experienced. During 12 weeks of treatment in total, 16 AEs were reported, six (10.2%) in the test product group, six (10.3%) in the reference product 1 group, and four (6.6%) in the reference product 2 group. No statistically significant difference was observed between the groups regarding AE occurrences (p=0.715).

**Table 2:** AEs reported by patients in different treatment arms AEs: Adverse events; UTI: Urinary tract infection. Test product (vildagliptin 50 mg + pioglitazone 15 mg FDC tablets), reference product 1 (vildagliptin 50 mg tablets), and reference product 2 (pioglitazone hydrochloride 15 mg tablets)

	Test product (n=59)	Reference product 1 (n=58)	Reference product 2 (n=61)
AE event	n (%)		
Bilateral pedal edema	-	1 (1.72%)	-
Gastritis	1 (1.69%)	-	-
Vaginal itching	-	-	1 (1.63%)
Cough, cold, fever	-	1 (1.72%)	-
Severe abdominal pain and vomiting	-	-	1 (1.63%)
Abdominal pain and bloating	-	-	1 (1.63%)
UTI	1 (1.69%)		-
Diarrhea	-	1(1.72%)	-
Loss of appetite	1 (1.69%)	-	-
Dengue	-	2 (3.45%)	-
Fever	-	1(1.72%)	1(1.63%)
Foot burn	3 (5.08%)	-	-
Total AEs	6	6	4

All reported AEs in the test and reference product 1 group were classified as mild and not suspected to be related to the study medication. In the reference product 2 group, 2 (3.3%) were of mild severity, and 2 (3.3%) were of moderate severity. Two (3.3%) patients in the reference product 2 group discontinued their medication due to AE, while none discontinued in the other groups. A significantly higher proportion of patients with AE in the test product required no action compared to other groups (p = 0.016). No serious adverse events (SAEs) or deaths were reported during the study. The lipid profile, kidney function test, hematological parameters, and body weight did not show significant changes over time. Liver enzymes ALT reduced significantly at week 12 in the test and reference product 2 groups (p = 0.029 and 0.023, respectively). At the same time, significant reductions were seen in alkaline phosphatase in the reference product 2 group (p = 0.041).

## Discussion

This 12-week, randomized study found that in Indian T2DM patients who were uncontrolled on metformin monotherapy, vildagliptin, and pioglitazone FDC significantly lowered HbA1c, FPG, and 2hr-PPG compared to component monotherapy. It is established that monotherapies' failure to act on the multiple pathophysiological mechanisms involved in T2DM and the progressive deterioration of β-cell function justifies the early use of combination therapy with different classes of drugs [12].

Combining vildagliptin and pioglitazone is a promising and logical therapeutic approach [13]. As a distinct mechanism of action, the vildagliptin and pioglitazone combination provides better glycemic control than either monotherapy component [13]. Similar to previous research, this study showed a statistically significant reduction in HbA1c with vildagliptin and pioglitazone FDC, and the proportion of patients achieving HbA1c < 7% was also higher than in either monotherapy. Furthermore, FPG and 2hr-PPG decreased numerically and statistically more with the vildagliptin and pioglitazone FDC than with the individual component monotherapy. Besides this, combining DPP-4 inhibitors with pioglitazone is generally well tolerated [7]. As established in this study, which demonstrated good tolerability of vildagliptin and pioglitazone FDC with no SAEs. Moreover, the glucose-dependent effect of vildagliptin, i.e., absence of absolute hyperinsulinemia, explains the very low incidence of hypoglycemia seen with the vildagliptin and pioglitazone combination despite the threefold increase in insulin secretion rate adjusted for glucose [14]. Likewise, very few hypoglycemic events were reported in this study.

Several other clinical trials have revealed that a combination of vildagliptin and pioglitazone exhibits beneficial effects. A 24-week, randomized, double-blinded study at 145 centers in eight countries showed first-line treatment with vildagliptin and pioglitazone combination (high 30/100 mg and low dose 15/50 mg) provided better glycemic control than each component monotherapy with minimal hypoglycemia. Most prominently, even with a reasonably high baseline HbA1c (> 8% in 70% of patients), the high-dose combination allowed 65% of patients to achieve the recommended target level of HbA1c of < 7.0% with negligible hypoglycemia (1%) [13].

In a 24-week, multicenter, double-blind, randomized, parallel-group study by Garber et al., a highly selective DPP-4 inhibitor vildagliptin was added to a maximally effective therapeutic dose of the pioglitazone improved glycemic control and was well tolerated in patients with T2DM [15]. A post hoc analysis reported that a high-dose combination of vildagliptin and pioglitazone (30/100 mg) improved glycemic control with significant decreases in HbA1c, PPG, and FPG, respectively, as well as significantly higher HbA1c response rates compared with pioglitazone monotherapy at 24 weeks.

The combination was well tolerated, with similar rates of AEs and SAEs in all treatment groups and with no obvious link between treatment and dose [3].

Interestingly, this study found that vildagliptin and pioglitazone FDC significantly reduced ALT levels at 12 weeks. It has been demonstrated that liver enzymes, especially ALT, are independently associated with nonalcoholic fatty liver disease in T2DM [15]. Hence, these findings suggest a possible role of vildagliptin and pioglitazone FDC in nonalcoholic fatty liver disease in T2DM patients.

Our study has some limitations. Since it was an open-label study, reporting bias cannot be excluded. In addition, we have not evaluated the responder rate for HbA1c, PPG, and 2hPPG. Furthermore, though HbA1c values differed significantly between all three groups, we could not explain the nonsignificant difference in the percentage of patients who achieved HbA1c < 7%. This could be related to the baseline characteristics response and short time frame.

## Conclusions

Vildagliptin and pioglitazone FDC are more effective than vildagliptin and pioglitazone monotherapy in managing T2DM patients inadequately controlled on metformin. Treatment with vildagliptin and pioglitazone FDC showed significant reductions in all glycemic parameters, viz., HbA1c, PPG, and FPG compared to vildagliptin and pioglitazone monotherapy. Lipid parameters including TC, LDL, and HDL were measured at screening and at the completion of 12 weeks, and, as the study duration is short, there is no significant differences were found between study groups. The vildagliptin and pioglitazone FDC were well-tolerated, with a very low incidence of AEs and no incidence of SAEs. Thus, vildagliptin and pioglitazone FDC present an efficacious and well-tolerated addition to T2DM management in Indian patients.

Long-term randomized trials in a larger population are needed to further validate the efficacy and safety of vildagliptin and pioglitazone FDC. Clinical studies are also required to evaluate the effect of vildagliptin and pioglitazone FDC on cardiovascular endpoints and its impact on the course of diabetes and β-cell function. Additionally, direct comparison with various drug combinations is needed to aid in the identification of the most effective and safe therapeutic approach for T2DM. Likewise, studies are required to evaluate the potential of vildagliptin and pioglitazone FDC in nonalcoholic fatty liver disease in diabetes patients.
